# Monocarboxylate Transporter 8 Modulates the Viability and Invasive Capacity of Human Placental Cells and Fetoplacental Growth in Mice

**DOI:** 10.1371/journal.pone.0065402

**Published:** 2013-06-12

**Authors:** Elisavet Vasilopoulou, Laurence S. Loubière, Heike Heuer, Marija Trajkovic-Arsic, Veerle M. Darras, Theo J. Visser, Gendie E. Lash, Guy S. Whitley, Christopher J. McCabe, Jayne A. Franklyn, Mark D. Kilby, Shiao Y. Chan

**Affiliations:** 1 School of Clinical and Experimental Medicine, College of Medical and Dental Sciences, University of Birmingham, Birmingham, United Kingdom; 2 Leibniz Institute for Age Research/Fritz Lipmann Institute, Jena, Germany; 3 Laboratory of Comparative Endocrinology, Katholieke Universiteit, Leuven, Belgium; 4 Erasmus Medical Center, Rotterdam, The Netherlands; 5 Reproductive and Vascular Biology Group, Institute of Cellular Medicine, Newcastle University, Newcastle upon Tyne, United Kingdom; 6 Division of Biomedical Sciences, St George’s University of London, London, United Kingdom; VU University Medical Center, The Netherlands

## Abstract

Monocarboxylate transporter 8 (MCT8) is a well-established thyroid hormone (TH) transporter. In humans, MCT8 mutations result in changes in circulating TH concentrations and X-linked severe global neurodevelopmental delay. MCT8 is expressed in the human placenta throughout gestation, with increased expression in trophoblast cells from growth-restricted pregnancies. We postulate that MCT8 plays an important role in placental development and transplacental TH transport. We investigated the effect of altering MCT8 expression in human trophoblast *in vitro* and in a Mct8 knockout mouse model. Silencing of endogenous MCT8 reduced T3 uptake into human extravillous trophoblast-like cells (SGHPL-4; 40%, P<0.05) and primary cytotrophoblast (15%, P<0.05). MCT8 over-expression transiently increased T3 uptake (SGHPL-4∶30%, P<0.05; cytotrophoblast: 15%, P<0.05). Silencing MCT8 did not significantly affect SGHPL-4 invasion, but with MCT8 over-expression T3 treatment promoted invasion compared with no T3 (3.3-fold; P<0.05). Furthermore, MCT8 silencing increased cytotrophoblast viability (∼20%, P<0.05) and MCT8 over-expression reduced cytotrophoblast viability independently of T3 (∼20%, P<0.05). *In vivo*, Mct8 knockout reduced fetal:placental weight ratios compared with wild-type controls at gestational day 18 (25%, P<0.05) but absolute fetal and placental weights were not significantly different. The volume fraction of the labyrinthine zone of the placenta, which facilitates maternal-fetal exchange, was reduced in Mct8 knockout placentae (10%, P<0.05). However, there was no effect on mouse placental cell proliferation *in vivo*. We conclude that MCT8 makes a significant contribution to T3 uptake into human trophoblast cells and has a role in modulating human trophoblast cell invasion and viability. In mice, Mct8 knockout has subtle effects upon fetoplacental growth and does not significantly affect placental cell viability probably due to compensatory mechanisms *in vivo*.

## Introduction

The importance of maternal thyroid hormone (TH) availability to normal fetoplacental development is highlighted by the association of untreated maternal thyroid dysfunction with pregnancy complications, including miscarriage, pre-eclampsia, intrauterine growth restriction (IUGR) and stillbirth [1], [2], [3], [4]. These complications are often characterized by malplacentation or uteroplacental insufficiency. Such associations are therefore postulated to occur through the direct effects of maternal TH upon placental development, as well as through alterations in the transplacental passage of TH from the mother to the fetus.

The human placenta is thought to be TH-responsive [5], as evidenced by the expression of the full complement of proteins that are required to mediate TH action from early gestation onwards. The entry of maternal TH into trophoblast, as well as its passage across the placenta to the developing fetus, is facilitated by several plasma membrane proteins that can transport THs, including the monocarboxylate transporter 8 (MCT8) [6], [7]. From early gestation the human placenta also expresses TH receptor isoforms (TRα1, TRβ1), which are nuclear transcription factors that can bind to the active TH ligand, tri-iodothyronine (T3), to regulate transcription of TH-responsive genes [8], [9]. Furthermore the human placenta demonstrates pre-receptor regulation by deiodinase enzymes type 2 (D2; converts thyroxine [T4] to T3) and type 3 (D3; inactivates T4 and T3) [10] with the latter playing a critical role in regulating the transplacental passage of TH [11].

The *SLC16A2* gene located on the X chromosome encodes the MCT8 protein, a 63 kDa transmembrane protein, which is a well-established TH transporter. Injection of rat Mct8 mRNA into *Xenopus* oocytes resulted in a 10-fold increase in T4 and T3 uptake [12], whilst the transfection of human MCT8 cDNA into COS1 and JEG3 cells, which have little or no endogenous MCT8, more than doubled the uptake of T3 [13]. MCT8 has a broad tissue distribution, with the placenta being one of the tissues showing a relative abundance of MCT8 expression [14], [15]. There has been much interest in this gene since the discovery that human MCT8 mutations result in X-linked severe global neurodevelopmental delay associated with low circulating T4, high T3 and normal or slightly elevated thyroid stimulating hormone (TSH) [16], [17], [18], [19]. Although it is widely believed that impairments in MCT8 function within the fetal brain, liver and other organs result in this phenotype [20], it is still unknown if disrupted transplacental passage of TH or impaired placental development also contribute to the phenotype.

Recently, two genotypically different Mct8 knockout mice models have been developed [21], [22]. Both models demonstrate changes in serum TH concentrations similar to that observed in patients affected by MCT8 mutations. Unlike human patients, Mct8 knockout mice do not exhibit any overt neurodevelopmental disorders nor a decline in postnatal growth. There is also no difference in fertility or reproductive function. However, the effect of Mct8 deficiency on fetoplacental growth and development has not been investigated so far.

We have previously reported that MCT8 is expressed in the human placenta from six weeks of gestation with increased expression with advancing gestational age [6], [23]. Within the human placenta MCT8 protein is localized to villous cytotrophoblast, syncytiotrophoblast (a layer formed by differentiating cytotrophoblast cells fusing together that constitutes the primary physical barrier that controls transplacental transport) and extravillous trophoblast (EVT; cells that invade into the decidualized maternal endometrium and spiral arteries to facilitate adequate maternal blood supply into the placental bed) [23]. MCT8 protein expression in the human placenta, and specifically in primary villous cytotrophoblast, is significantly increased in pregnancies complicated by IUGR compared with gestationally-matched appropriately grown controls [6], [23], [24]. *In vitro* experiments have demonstrated that T3 increases the invasive potential of primary EVT [25] and suppresses their apoptosis [26], and could promote epidermal growth factor production by first trimester placental explants [27].

We hypothesized that MCT8 could influence placental development by regulating TH uptake and hence the intracellular availability of T3, which alters human trophoblast cell function and viability. To investigate this hypothesis, MCT8 expression was silenced or over-expressed in two human trophoblast cell types: EVT and cytotrophoblast cells. In addition, the Mct8 knockout mouse model was used to investigate the role of MCT8 in fetoplacental growth and placental morphology *in vivo*.

## Materials and Methods

### *In vitro* Experiments

#### Ethical approval

Human samples were collected with informed written consent and with the approval of the South Birmingham research ethics committee and the Research and Development office of the Birmingham Women’s NHS Foundation Trust according to the Declaration of Helsinki.

#### Sample collection

Human placentae from uncomplicated pregnancies were collected following elective pregnancy termination (n = 5; 11 weeks of gestation) or elective caesarean section (n = 13; median gestational age 39 weeks, range 38^+3^–39^+2^). The fetuses were not known to have abnormal karyotypes and none of the pregnancies was complicated by thyroid disorders.

#### Cell isolation and culture

To investigate the role of MCT8 in EVT, primary EVT cells were isolated from first trimester human placentae, as described previously [28] and cultured on a thin layer of growth factor-reduced Matrigel® matrix (BD Biosciences, Erembodegem, Belgium). Primary EVT cells were used for initial experiments. Due to the limited cell numbers obtainable from first trimester placenta, a cell line derived from primary EVT, SGHPL-4 [29], was used for further studies. The role of MCT8 in villous cytotrophoblast cells was investigated using primary cytotrophoblast cells isolated from human term placentae, as described previously [8], [24], [30], [31]. All media were supplemented with either 10% (v/v) fetal calf serum (FCS; Invitrogen, Paisley, UK) or with 10% (v/v) charcoal-stripped fetal calf serum (SFCS; First Link, Birmingham, UK), which is devoid of THs, for experiments that involved T3 treatment.

#### Silencing and over-expression of MCT8 expression

Amaxa® electroporation (Lonza, Basel, Switzerland) was used for the delivery of small interfering RNA (siRNA) or plasmid DNA into cells as per manufacturer’s instructions. For SGHPL-4 cells, solution V and program X-001 were used. The kit for primary epithelial cells and program T-013 was used for cytotrophoblast cells. For the suppression of endogenous MCT8 expression (MCT8 silencing), cells were transfected with MCT8-specific siRNA (final concentration 100 nM; sense sequence: GCCUGCGCUACUUCACCUAtt) or with a non-targeting control (sense sequence: GGGCCACAGUUUCAGCUUCtt; Ambion, Warrington, UK). For transient up-regulation of MCT8 expression (MCT8 over-expression) the cells were transfected with plasmid DNA (1 µg) (pcDNA3.1+; Invitrogen) containing the cDNA for human wild-type MCT8 with a C-terminal hemagglutinin (HA) tag [32]. PcDNA3.1+ plasmid lacking the MCT8 cDNA was used as a vector only (VO) control. Cell recovery, assessed by cell counting under a microscope 24 hours post-transfection, was approximately 80% for SGHPL-4 and 50% for cytotrophoblast (data not shown).

Successful silencing of endogenous MCT8 and overexpression of HA-tagged MCT8 were confirmed by TaqMan PCR, flow cytometry and western blotting, as previously described [24]. In SGHPL-4 cells, transfection with MCT8 siRNA resulted in a 70% reduction in MCT8 mRNA (P<0.001) with undetectable MCT8 protein in lysates from cells transfected with MCT8 siRNA (Figures S1A–B). Over-expression of MCT8 protein 72 hours post-transfection assessed by flow cytometry showed that 41% of SGHPL-4s were expressing the transfected MCT8 protein and increased protein expression was also confirmed by western blotting (Figure S1C–D). In cytotrophoblast cells, transfection with MCT8 siRNA resulted in 90% reduction in MCT8 mRNA expression (P<0.001) and undetectable MCT8 protein (Figure S2A–B). Over-expression of MCT8 protein assessed by flow cytometry showed that 28% of cytotrophoblast cells were expressing the transfected MCT8 protein and this was also confirmed by western blotting (Figure S2C–D).

#### T3 uptake assays

Following silencing or over-expression of MCT8 expression the cells were cultured for 72 hours in duplicate in 10% FCS-supplemented medium. The cells were incubated with serum-free medium supplemented with 1 nM T3 containing approximately 2×10^5 ^cpm of [^125^I]-T3 (Perkin Elmer, Massachusetts, USA) and [^125^I]-T3 uptake was measured, as previously described [24]. In addition, SGHPL-4 cells were also transfected with plasmid containing the μ-crystallin cDNA (CRYM; Imagenes; Berlin, Germany), on its own or co-transfected with MCT8. Since CRYM binds T3 intracellularly [33], [34], co-transfection with this protein was used to increase the intracellular T3-binding capacity of the cells and thus decrease the rate of T3 efflux.

#### Cell invasion

Following isolation (primary EVT) or Amaxa® electroporation (SGHPL-4), cells were cultured in cell culture inserts [8 µm membrane pore size] coated with 10 µl of growth factor-reduced Matrigel® matrix (BD Biosciences). Cells were treated with 0 or 10 nM T3 and cell invasion through Matrigel® was assessed 48 or 72 hours later by counting all the invaded cells visualized with Mayer’s hematoxylin and eosin (H&E). The invasion index was determined and expressed as the ratio of invaded cells in the experimental group relative to that in the respective control group (EVT: cells treated with 0 nM T3; SGHPL-4: cells transfected with control siRNA or VO and treated with 0 nM T3).

#### Cell motility

The effect of MCT8 silencing or over-expression on SGHPL-4 motility was assessed. To identify cells that were successfully transfected with MCT8, a plasmid containing cDNA encoding a fusion protein for hMCT8 and GFP was constructed (MCT8-GFP). PcDNA3.1+ plasmid encoding GFP was used as control (GFP). Transfection of SGHPL-4 with MCT8-HA or MCT8-GFP resulted in a similar increase in T3 uptake, which was 30% (±7) and 30% (±13) respectively following incubation with [^125^I]-T3 for 10 minutes. Following electroporation the cells were allowed to adhere overnight in 10% (v/v) FCS-supplemented media followed by a 4-hour incubation in media supplemented with 10% (v/v) SFCS before treatment with 0 or 10 nM T3. Cell motility was assessed by time-lapse microscopy (TLM) over 20 hours, as described previously [29]. Twenty cells per field of view were chosen at random and the distance moved was measured using ImageJ (U. S. National Institutes of Health, Bethesda, MD, USA). Within each experiment the results were normalized to the values obtained from the control groups (as defined above).

#### Cell survival and apoptosis

SGHPL-4 cells were allowed to adhere overnight in 10% (v/v) FCS-supplemented media followed by a 4-hour incubation in media supplemented with 10% (v/v) SFCS before treatment with 0 or 10 nM T3 for 48 hours. Primary term cytotrophoblast cells were cultured in media supplemented with 10% (v/v) SFCS and from 18 hours post-isolation were treated with 0 or 10 nM T3 for 48 hours. Cell number was assessed using the methyltetrazoleum (MTT; Sigma-Aldrich) colorimetric assay as described previously [8] and by counting cell nuclei stained with Hoechst 33258 (1∶1000; Sigma-Aldrich). In addition, cell apoptosis was assessed using the luminescence-based Caspase 3/7 activity assay (Promega, Southampton, UK) [32]. The results were normalized to the values obtained from the control groups (as defined above) within each experiment. Caspase 3/7 results were also normalized to the MTT results to correct for cell numbers. In addition, apoptosis in SGHPL-4 cells was assessed based on changes in cell morphology seen by TLM as previously described [35].

### *In vivo* Experiments in a Murine Model

#### Matings, euthanisation and sample collection

Animal studies were approved by the Animal Welfare Committee of the Medizinische Hochschule Hannover, Germany. Female Mct8^+/−^ mice were obtained from Deltagen (San Mateo, CA, USA). All animals were on a C57/Bl6 background, kept at constant temperature (22°C) and light cycle (12 hours light, 12 hours dark) and provided with standard laboratory chow and tap water *ad libitum*. Heterozygous female carrier mice (Mct8^+/−^; n = 26) were mated with male knockout (Mct8^−/y^) or wild-type mice (Mct8^+/y^). Pregnant dams were euthanized at gestational day 14 (E14.5; n = 17) or at gestational day 18 (E18.5; n = 9) by CO_2_ administration. Each fetus and placenta was dissected out separately and weighed. Fetal tails were genotyped by PCR to determine the sex (SRY gene) and Mct8 status, as previously described [22]. Fetuses were snap-frozen for analysis of TH content. Half of each placenta was fixed [4% (w/v) paraformaldehyde, 3% (w/v) PIPES, 3% (w/v) PVP40 and 0.02% (w/v) CaCl_2_ fixative (pH 7.4)] and paraffin-embedded and the other half was snap-frozen. Only male Mct8 knockout fetuses and placentae and male wild-type littermates were used for subsequent experiments.

#### Mct8 mRNA expression in mouse placenta

To demonstrate Mct8 expression in the mouse placenta, placentae of wild-type fetuses at E14.5 (n = 10) and E18.5 (n = 11) were assessed by TaqMan PCR, as previously described [24], using a commercially available set of primers and probe (Assay ID: Mm00486204_m1; Applied Biosystems, Paisley, UK).

#### Stereo-histological assessment of the placenta

Paraffin-embedded placentae from E18.5 Mct8^−/y^ and Mct8^+/y^ littermates (n = 6) were entirely sectioned into 7 µm-thick sections. Approximately every tenth section ranging from the centre to the periphery of the placenta (20 sections per placenta) was stained with H&E and photographed using a 2× objective lens. The volume fraction of each region of the mouse placenta [chorionic plate, labyrinthine zone (Lz), junctional zone, decidua basalis] was estimated using a grid, as previously described [36]. Absolute placental volumes were calculated by multiplying the wet placental weight of each sample by 1.05 (the estimated specific gravity of placenta) [37].

#### TH measurements in fetuses

In order to assess maternal to fetal transfer of TH, the total fetal T4 and T3 content at E14.5 (before endogenous fetal TH production) was measured by radio-immuno assay following TH extraction from tissues, as described in detail previously [38]. Two to three fetuses of the same genotype from each litter were pooled together (2–3 per genotype) for the assay. However, the levels of T4 and T3 were below the detection limits of the assay in all the samples, both wild-type and Mct8 knockout.

#### Deiodinase activity assays

The activity of D2 and D3 in wild-type and Mct8 knockout mouse placentae at E14.5 was assessed by HPLC, as described previously [39]. From each litter (n = 7), three placentae of each genotype were pooled together and processed as a single sample.

#### Cell proliferation in the mouse placenta

To compare cell proliferation between wild-type and Mct8 knockout placentae, the protein expression of Cyclin D1 (1/200; ab16663, Abcam, Cambridge, UK) in whole placental homogenates (E18.5; n = 6 litters) was assessed by western blotting, as described previously [24]. To isolate potential changes in trophoblast viability specifically within the Lz, we counted the number of endothelial (CD31+ve; stained with antibody ab56299; 1/100; Abcam) and trophoblast (CD31-ve and identified also by characteristic cell morphology) cells (all counterstained with Mayer’s hematoxylin) in five randomly selected fields per placental section (viewed under a 20× objective) from wild-type and knockout placentae (E18.5: n = 4 litters).

### Statistical Analysis

Data were analyzed using Minitab® statistical software (version 15). When comparing two groups, statistical significance was assessed by paired t-test. For comparisons of more than two groups, or groups affected by more than one variable, analysis of variance (ANOVA) was performed using the general linear model. Tukey all pairwise multiple comparisons post-hoc tests were used to assess differences between individual groups. Residuals for all data sets passed the normality test as determined using the Kolmogorov-Smirnov test.

## Results

### Effect of MCT8 on T3 Uptake *in vitro*

**Figure 1:**
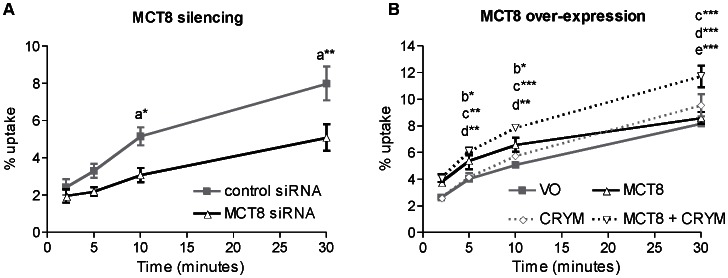
T3 uptake by SGHPL-4 cells following MCT8 silencing (A) or MCT8 over-expression (B). SGHPL-4 cells were incubated for 0 to 30 minutes with [^125^I]-T3. The amount of intracellular radioactivity was quantified and expressed as a percentage of the total radioactivity added. Points represent average of three experiments ±SEM. Within each experiment, each condition was performed in duplicate. Statistically significant differences are indicated by ^a^(control siRNA *vs* MCT8 siRNA) ^b^(VO *vs* MCT8), ^c^(VO *vs* MCT8+ CRYM), ^d^(MCT8+ CRYM *vs* CRYM) and ^e^(MCT8 *vs* MCT8+ CRYM); *P<0.05, **P<0.01 and ***P<0.001.

To assess the contribution of endogenous MCT8 to TH transport, we assessed T3 uptake by the EVT-like cell line, SGHPL-4, and primary term human cytotrophoblast cells following MCT8 silencing. MCT8 silencing was associated with a significant reduction in T3 uptake by SGHPL-4 cells (Figure 1A) with a maximum effect at 10 minutes (40% reduction, P<0.05). The inhibition of T3 uptake was sustained for the entire 30 minutes of incubation with [^125^I]-T3. Conversely, MCT8 over-expression increased T3 uptake by SGHPL-4 cells (Figure 1B; 30%; P<0.05) at 10 minutes. However, at 30 minutes net T3 uptake was similar in MCT8-transfected cells and in VO controls, presumably because equilibrium was reached by that point. This supports previous observations indicating that MCT8 could also facilitate T3 efflux as well as influx. This was confirmed when SGHPL-4 cells were co-transfected with MCT8 and CRYM, a cytosolic protein that binds to T3 intracellularly and thus decreases T3 efflux [33], [34]. T3 uptake by SGHPL-4 cells co-transfected with MCT8 and CRYM was significantly higher than by those transfected with VO (by 44%), or MCT8 alone (by 44%) or CRYM alone (by 28%) following 30 minutes of incubation with [^125^I]-T3 (P<0.001).

**Figure 2:**
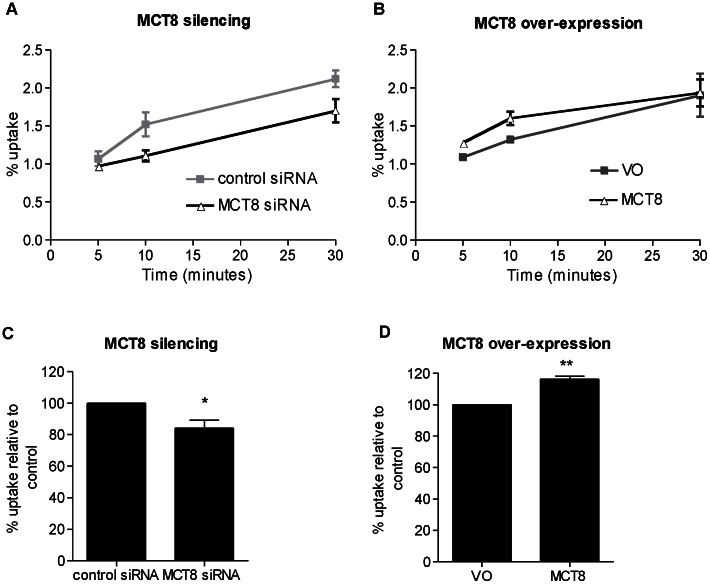
T3 uptake in primary cytotrophoblast cells following MCT8 silencing (A,C) or MCT8 over-expression (B,D). **A and B**: T3 uptake time course from one representative experiment. Cytotrophoblast cells were incubated for 0 to 30 minutes with [^125^I]-T3. The amount of intracellular radioactivity was quantified and expressed as a percentage of the total radioactivity added. **C and D**: Relative T3 uptake following incubation for 10 minutes with [^125^I]-T3. Bars represent average of four experiments +SEM. Within each experiment, each condition was performed in duplicate. The mean uptake for the control within each experiment has been assigned the value of 100%. Statisticallysignificant differences are indicated by *P<0.05 and **P<0.01.

In primary human cytotrophoblast cells T3 uptake over 30 minutes showed a similar pattern to that in SGHPL-4 cells. In initial time course experiments, MCT8 silencing resulted in reduced T3 uptake after 10 and 30 minutes of incubation with [^125^I]-T3 (Figure 2A), whilst MCT8 over-expression increased T3 uptake by primary cytotrophoblast cells only transiently with the maximum effect observed at 10 minutes (Figure 2B). To confirm these observations, further experiments were conducted with assessments made at 10 minutes (n = 4) showing that MCT8 silencing resulted in a 15% reduction in T3 uptake (Figure 2C, P<0.05), whilst MCT8 over-expression resulted in a 15% increase in T3 uptake by cytotrophoblasts (Figure 2D; P<0.01).

### Effect of MCT8 on Extravillous Trophoblast Invasion and Motility *in vitro*

**Figure 3:**
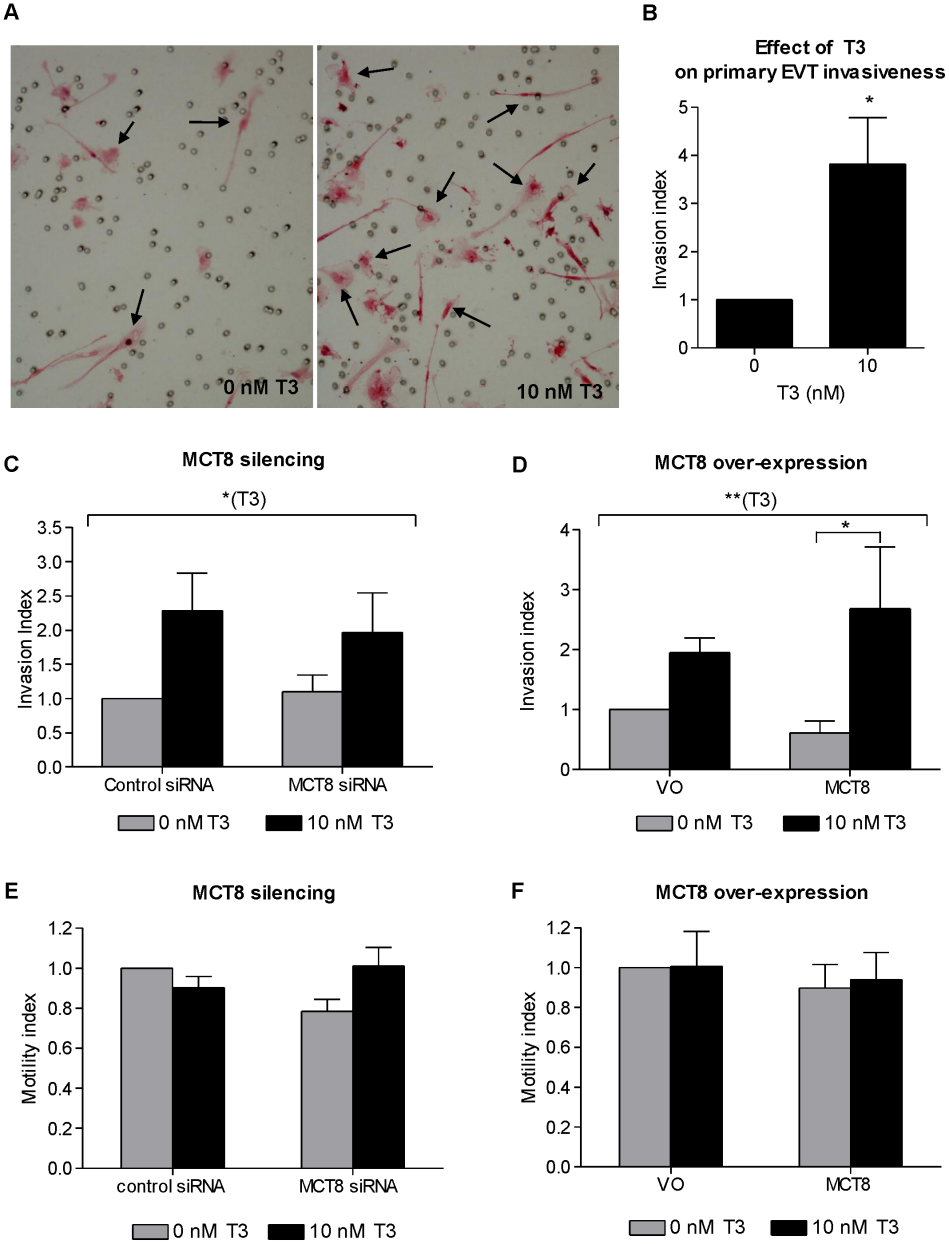
Effect of MCT8 and T3 on EVT invasiveness and motility. **A**: Representative pictures of first trimester primary EVT cells that invaded through Matrigel® (arrows) following treatment with 0 or 10 nM T3. The cells were stained with Mayer’s hematoxylin and eosin. **B**: Index of invasion by first trimester primary EVT following treatment with 0 or 10 nM T3. Bars represent average of six experiments+SEM. Within each experiment, each condition was performed in triplicate and the results normalized to the mean value for the controls. **C and D**: Invasion of the EVT-like SGHPL-4 cells through Matrigel® following silencing of endogenous MCT8 expression (C) or over-expression of MCT8 (D) and treatment with 0 or 10 nM T3. The number of invading cells was normalized to the average number of invading cells in the control groups that were transfected with control siRNA or VO and treated with 0 nM T3. Bars represent average of seven (silencing) or six (over-expression) experiments +SEM. Within each experiment, each condition was performed in duplicate. **E and F**: Motility of SGHPL-4 cells. Distance moved following MCT8 silencing with siRNA (E) or over-expression of GFP-tagged MCT8 (F) followed by treatment with 0 or 10 nM T3 for 20 hours. The distance over which cells moved was normalized to the average distance covered by cells in the control groups that were transfected with control siRNA or GFP only (VO) and treated with 0nM T3. Bars represent average of three experiments +SEM. Statistically significant differences are indicated by *P<0.05 and **P<0.01. The longer brackets indicate the overall effect of T3 across all groups by ANOVA and the shorter bracket indicates the significant difference between specific groups assessed by post-hoc testing.

Treatment of first trimester primary EVT cells with 10 nM T3 resulted in an increase in cell invasion (Figure 3A), with a mean increase of 3.8-fold compared with untreated cells (Figure 3B, P<0.05), which is consistent with previous reports [25]. To investigate the underlying mechanisms of this observation and the effect of altered MCT8 expression on cell invasion we utilized the EVT-like cell line, SGHPL-4, as a model of EVT because of the limited cell numbers obtainable from first trimester placentae. Treatment of SGHPL-4 cells with 10 nM T3 also resulted in an overall trend of increased cell invasion (ANOVA effect of T3 P<0.05), suggesting that this is a valid cell model to study T3-mediated effects on EVT invasion (Figures 3C–D). Even though silencing MCT8 had no effect on SGHPL-4 invasion through Matrigel® (Figure 3C), with over-expression of MCT8 T3 induced a significant pro-invasive effect resulting in a 3.3-fold increase in cell invasion compared with no T3 (P<0.05), which is more than the T3-induced effect when cells were transfected with VO (Figure 3D). In contrast, SGHPL-4 motility was not affected by changes in MCT8 expression or by T3 treatment (Figures 3E–F).

### Effect of MCT8 on Trophoblast Cell Viability *in vitro*

**Figure 4:**
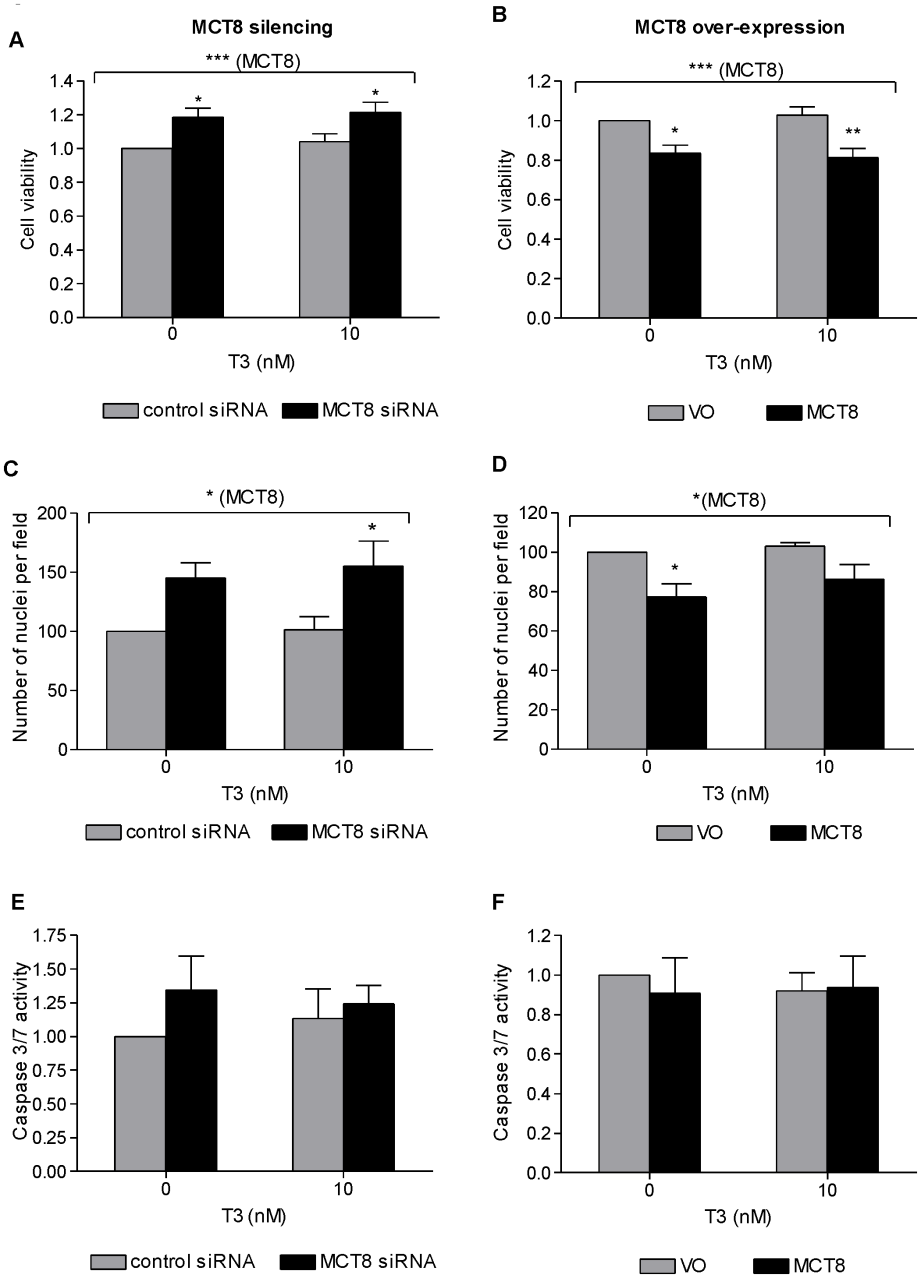
Effect of MCT8 silencing (A,C,E) and MCT8 over-expression (B,D,F) on primary term cytotrophoblast cell viability. assessed by MTT assay (A–B; average of six experiments +SEM, four replicates were performed per condition within each experiment) and by counting nuclei under a microscope (C–D; average of 3 experiments +SEM; within each experiment 5 fields were counted), and apoptosis by quantification of Caspase 3/7 activity (E–F; average of five experiments +SEM, within each experiment each condition was performed in triplicate). The assays were performed 72 hours following Amaxa electroporation, and 48 hours post-treatment with 0 or 10 nM T3. Results of each sample was normalized to the mean for cells transfected with control (control siRNA or VO) and treated with 0 nM T3. Caspase activity results were also normalized to the MTT results in order to correct for cell numbers. Statistically significant differences are indicated by *P<0.05, **P<0.01 and ***P<0.001.

Silencing endogenous MCT8 in primary term cytotrophoblast cells increased cell viability as assessed by MTT both in non-treated (19%, P<0.05) and T3-treated cells after 48 hours (16%, P<0.05; Figure 4A). Conversely, over-expression of MCT8 decreased cell viability by 17% in the absence of T3 (P<0.05) and 21% in the presence of T3 (P<0.01) (Figure 4B). This effect was also confirmed when the number of cell nuclei was counted. There were more nuclei following MCT8 silencing (ANOVA effect of MCT8: P<0.05; Figure 4C) and fewer nuclei following MCT8 over-expression (ANOVA effect of MCT8: P<0.05; Figure 4D), with similar trends observed in the absence and presence of T3. However, neither MCT8 silencing nor over-expression nor T3 treatment affected apoptosis measured by Caspase 3/7 activity (Figures 4E–F).

**Figure 5:**
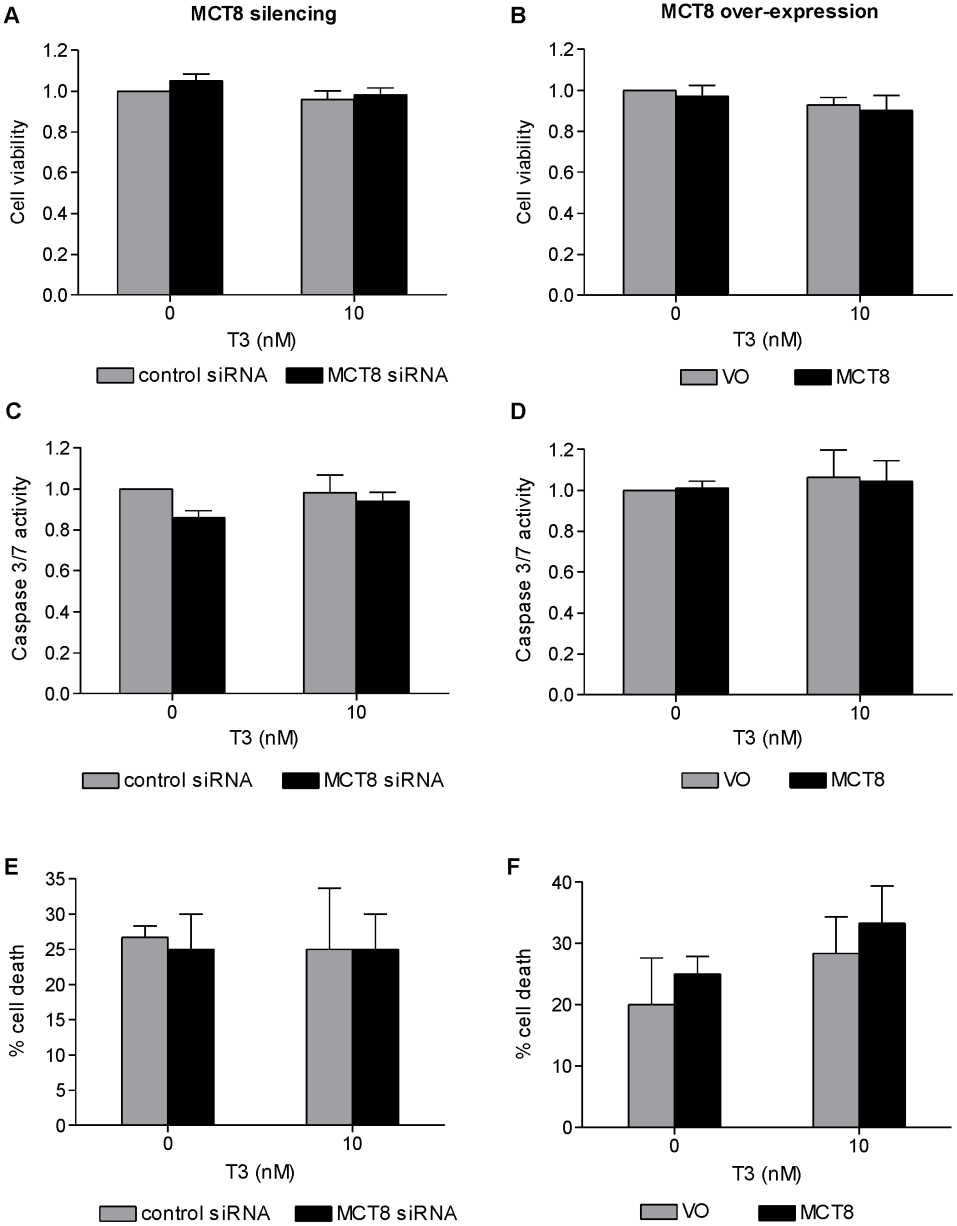
Effect of MCT8 silencing (A,C,E) and MCT8 over-expression (B,D,F) on the viability of the EVT-like SGHPL-4 cells. assessed by MTT assay (A–B), and apoptosis by quantification of Caspase 3/7 activity (C–D) and counting the number of apoptotic cells by time lapse microscopy (E–F). The assays were performed 72 hours following silencing of endogenous MCT8 or transfection with human wild-type MCT8, and 48 hours post-treatment with 0 or 10 nM T3. **A and B**: The absorbance for each sample was normalized to the average absorbance measured in cells transfected with control (control siRNA or VO) and treated with 0 nM T3. The bars represent average of five experiments +SEM. Four replicates were performed per condition within each experiment. **C and D**: Results were normalized to the MTT results in order to correct for cell numbers. Bars represent average of three experiments +SEM. Within each experiment, each condition was performed in triplicate. **E and F:** Bars represent average of three experiments +SEM. Within each experiment 20 cells were counted per condition.

In contrast to the observations in primary cytotrophoblast cells, both silencing and over-expression of MCT8 in SGHPL-4 cells, a model of EVT, did not affect cell viability as measured by MTT (Figure 5A–B). Like primary cytotrophoblasts, however, MCT8 also did not affect apoptosis of SGHPL-4 cells as measured by Caspase 3/7 activity and by morphological assessment by TLM (Figure 5C–F).

### Mct8 Expression in the Mouse Placenta

Messenger RNA encoding Mct8 was demonstrated in the wild-type mouse placenta at both E14.5 and E18.5. Mct8 mRNA expression as determined by quantitative RT-PCR was significantly increased by 4.7 (±1.2) fold (mean±SEM; P<0.01) at E18.5 compared with E14.5.

### Effect of Mct8 on Placental Deiodinase Activities in Mice

D2 activity was undetectable in the mouse placenta. There was no significant difference in D3 activity between Mct8 wild-type (177.8±37.26 fmol/mg/min; mean±SEM) and knockout (224.0±51.74 fmol/mg/min) placentae at E14.5.

### Effect of Mct8 on Mouse Fetal and Placental Weights

**Figure 6:**
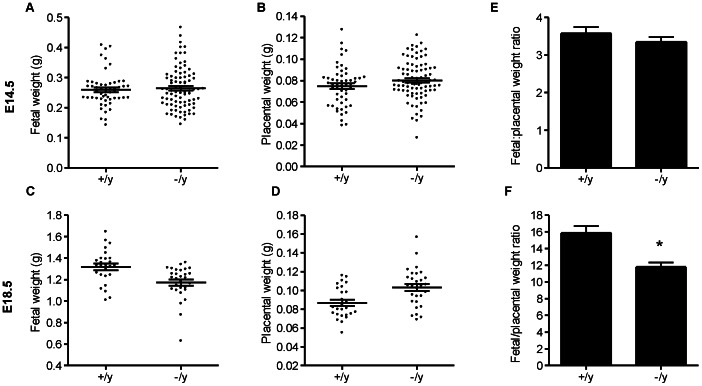
Absolute fetal (A and C), absolute placental (B and D) weights and fetal to placental weight ratios (E and F) of male wild-type (+/y) and male Mct8 knockout (−/y) fetuses at gestational day 14 (E14.5; n = 17 litters) and 18 (E18.5; n = 9 litters). Each dot represents the weight of individual samples and the lines represent the mean ±SEM for each group. Significant differences between groups are indicated by *P<0.05.

In order to find out if lack of Mct8 affects fetal growth or placental growth we recorded fetal and placental weights at two gestational ages, E14.5 (which is before the onset of fetal TH production) and E18.5 (which is after the onset of fetal TH production and just before delivery). At E14.5 there was no statistically significant difference in absolute fetal and placental weights between wild-type fetuses and Mct8 knockout littermates (Figure 6A–B). However, at E18.5 there was a trend of reduced fetal weights accompanied by a trend of increased placental weights with Mct8 knockout compared to wild-type (Figure 6C–D). This led us to calculate the ratio of fetal:placental weight for each conceptus, which gives an estimate of the fetal weight that is supported by each gram of placenta. We found that at E18.5 the fetal:placental weight ratio was 25% lower in Mct8 knockout fetuses (P<0.05), but there was no difference at E14.5 (Figure 6E–F). This is suggestive of placental inefficiency with Mct8 knockout at E18.5.

### Effect of MCT8 on Mouse Placental Morphology

**Figure 7:**
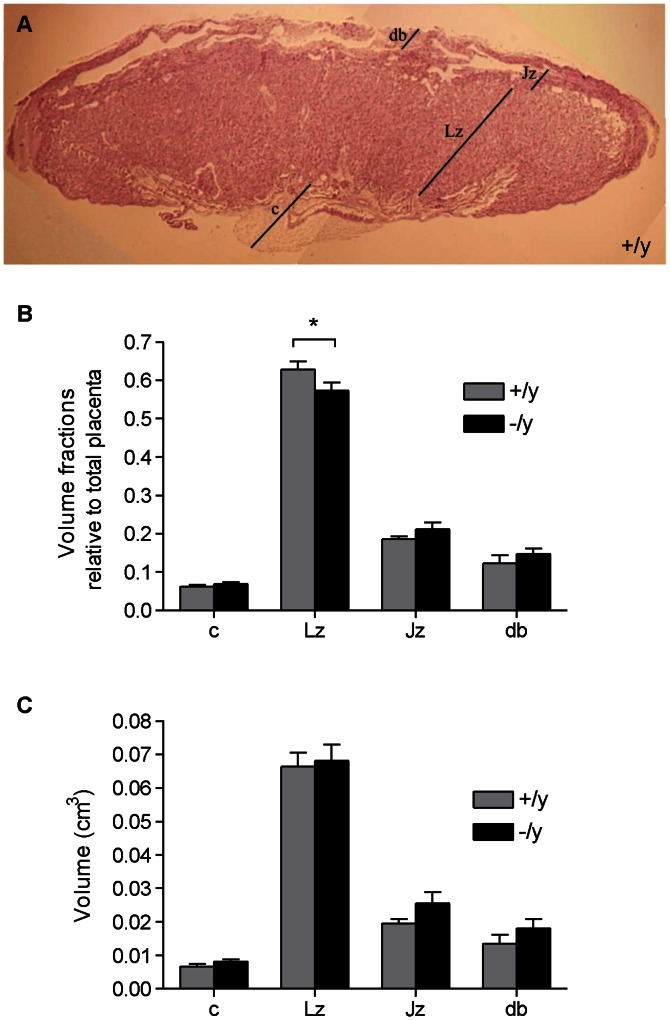
Stereohistological analysis of male wild-type (+/y) and male Mct8 knockout (−/y) E18.5 placentae. **A**: Representative picture of a placental section (wild-type), stained with hematoxylin and eosin, and photographed using a 2× objective lens. The different regions of the mouse placenta are annotated: db = decidua basalis, Lz = labyrinthine zone, Jz = junctional zone and c = chorionic plate. **B–C**: Volume fractions (B) of the placental regions relative to the whole placenta and absolute volumes (C) of the placental regions in wild-type and litter-matched Mct8 knockout samples. Six placentae were assessed per genotype. The bars represent the mean ±SEM for each group. Significant differences between groups are indicated by *P<0.05.

To look for other features indicating placental inefficiency we assessed whether placental morphology was affected at E18.5. We performed stereohistological analysis and calculated the volume fractions and the absolute volumes of each of the regions that make up the mouse placenta (Figure 7A). The volume fraction of the Lz relative to the whole placenta was smaller in Mct8 knockout samples compared to litter-matched wild-type samples (P<0.05) (Figure 7B). However, there was no difference in the absolute volume of the Lz between wild-type and knockout placentae (Figure 7C). No significant differences were observed when comparing the volume fraction or the absolute volume of the other regions in the mouse placenta, although there was a general trend of increased absolute volumes of all regions with Mct8 knock-out, with the Lz showing the least change.

### Effect of MCT8 on Cell Proliferation in the Mouse Placenta

**Figure 8:**
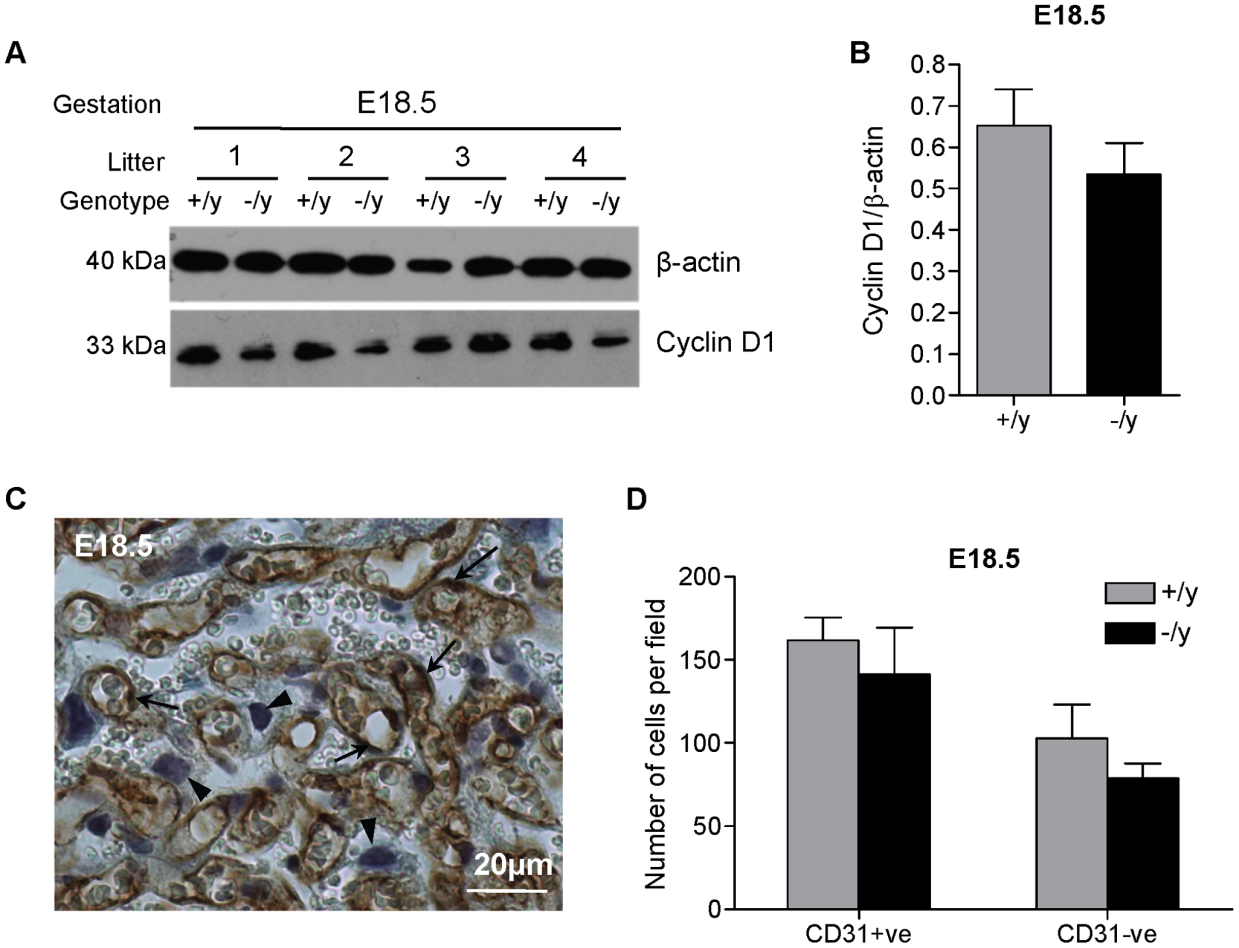
Proliferation and cell number in male wild-type (+/y) and male Mct8 knockout (−/y) placentae. (A): Changes in the protein expression of Cyclin D1 (proliferation) were assessed by western blotting. The expression of β-actin was assessed to control for sample loading. A representative blot is shown for gestational age E18.5. (B): Relative densitometry analysis based on one wild-type and one Mct8 knockout placenta from each of six litters at E18.5. The bars represent the mean ±SEM. (C): Representative picture demonstrating CD31 immunoreactivity (marker of endothelial cells represented by brown staining) in the mouse placenta at E18.5. The nuclei were counterstained using hematoxylin. Endothelial cells are indicated by arrows and trophoblast cells by arrowheads. (D): Number of trophoblast and endothelial cells (counted in five fields per sample under a 20× objective) in the labyrinthine zone of +/y and −/y placentae. One wild-type and one Mct8 knockout placenta from each of four litters at E18.5 were assessed. The bars represent the mean ±SEM.

Since *in vitro* findings suggest that the level of MCT8 expression affects the viability of human cytotrophoblast cells, we assessed proliferation in mouse wild-type compared with litter-matched Mct8 knockout placentae (Figure 8). In whole placental homogenates, there was no significant difference in the protein expression of the proliferation marker, Cyclin D1, at E18.5 (Figure 8A–B). In light of the findings in human trophoblast and in the mouse stereology, we sought to isolate possible differences in cell viability within the Lz. Cell counts of trophoblast and endothelial cells (distinguished by CD31 staining in conjunction with cell morphology; Figure 8C) were no different between wild-type and Mct8 knockout samples at E18.5. The average number of trophoblast cells per field (±SEM) was 103±20 in wild-type and 79±9 in Mct8 knockout placentae and the average number of endothelial cells per field was 162±13 and 141±28, respectively (Figure 8D).

## Discussion

In this study we have demonstrated that MCT8 makes a significant contribution to T3 uptake by human trophoblast cells *in vitro*. We have also shown that MCT8 promotes the pro-invasive effect of T3 on the EVT-like cell line, SGHPL-4, whilst it reduces the viability of primary term human cytotrophoblast cells in a T3-independent manner *in vitro*. *In vivo* data from Mct8 knockout mice reveal reduced fetal to placental weight ratios, with a smaller volume fraction of the Lz, where maternal-fetal exchange occurs. However, significant changes in placental cell growth could not be demonstrated *in vivo*.

A significant reduction in net T3 transport following silencing of endogenous MCT8 *in vitro* suggests that MCT8 contributes significantly to net T3 transport by human placental cells. This is consistent with our observation that MCT8 makes a substantial contribution to T3 uptake by microvillous membrane vesicles derived from term syncytiotrophoblast [7]. Human primary term cytotrophoblast [6], [24], primary EVT [6] and SGHPL-4 cells (unpublished data) express other TH transporters in addition to MCT8, including MCT10, system-L amino acid transporters (LAT1, LAT2 and their obligatory heterodimer CD98) and organic anion transporting polypeptides 1A2 and 4A1. These TH transporters could all facilitate both T3 influx and efflux in the absence of MCT8. Despite some transporters having a similar affinity for T3: MCT8 (*K*_m_ = 0.86 µM [40]), system-L (*K*_m_ = 0.8 µM [41]) and OATP4A1 (*K*_m_ = 0.9 µM [42]), these other TH transporters cannot completely compensate when MCT8 is silenced, resulting in reduced T3 retention intracellularly. On the other hand, when MCT8 was over-expressed at “supraphysiological levels”, there was an initial increase in T3 uptake, which could not be maintained over a prolonged (30 minute) incubation with [^125^I]-T3. This suggests that MCT8 over-expression could facilitate a transient rapid increase in T3 uptake but not the retention of T3 intracellularly due to T3 efflux taking place, as confirmed by our studies using co-transfection of MCT8 and CRYM in SGHPL-4 cells. It is probable that MCT8 also contributed significantly to this T3 efflux, as has been described in COS1 cells [43]. The relatively smaller magnitude of increase in T3 uptake in primary cytotrophoblast compared to SGHPL-4 cells could be partly attributed to the lower transfection efficiency for cytotrophoblast (28% vs 41%).

These findings raise the possibility that transplacental TH transport and placental TH content may be impaired in pregnancies where the fetus is affected by a MCT8 mutation. To date there are no data on fetal TH levels from human pregnancies, where the child (and therefore the placenta) was affected by a MCT8 mutation. Studies in adult mice have shown that global Mct8 deletion results in a hypothyroid state in some organs (brain) and a hyperthyroid state in others (liver, kidney) [21], [22], [44], depending on the TH transporters and deiodinases that are active in each tissue. We have demonstrated the expression of Mct8 in the mouse placenta. However, our attempts to quantify transplacental TH transport by Mct8 in mice before the onset of endogenous fetal TH production have been hindered by the low levels of T4 and T3 present in E14.5 mouse fetuses. Furthermore, the finding that D3 activity in the placenta remains unaltered when Mct8 is absent, suggests that either Mct8 knockout does not alter intracellular placental TH content or D3 activity remains unaltered in the face of an altered intracellular TH concentration. This is similar to reports that placental deiodinase activity was not affected in human cases of fetal hypothyroidism [39] and maternal hypothyroidism [45], as well as following *in vitro* T3 treatment of human primary cytotrophoblast cells [10].

MCT8 over-expression amplified the pro-invasive effect of T3, suggesting that MCT8 may play a role in regulating invasion by promoting T3 transport into EVT and therefore increasing intracellular T3 availability. However, this effect was modest and may not be unique to MCT8, and other TH transporters could have a similar effect. Surprisingly, despite MCT8 silencing resulting in a 40% reduction in net T3 uptake, SGHPL-4 invasion was not affected significantly. It is possible that regulators of TH action, namely other TH transporters, D2, D3 and TRs compensate for the lack of MCT8. Yet we have found that T3 treatment of SGHPL-4 cells does not affect the expression of these genes (unpublished data). It is likely that in EVT there are other more dominant factors involved in the regulation of invasion, which could attenuate the effects of T3 insufficiency, such as autocrine and paracrine effects of trophoblast-secreted cytokines and growth factors. For example, insulin-like growth factor (IGF)-II has been shown to promote the invasion of EVT-like cells in an autocrine manner [46]. The lack of impact of MCT8 silencing upon trophoblast invasion is also reflected by the limited epidemiological data derived from the relatively few cases of MCT8 mutations in humans, with approximately 80 families identified world-wide thus far [47]. There have not yet been any reports of a preponderance of malplacentation disorders which are typically associated with impaired EVT invasion, such as IUGR, pre-eclampsia, preterm delivery and pregnancy loss, which suggests that compensatory mechanisms may exist *in vivo* in human to attenuate the effects of reduced T3 uptake by trophoblast cells.

There is already evidence that MCT8 regulates cell turnover in several cell types. Reports of a hyperplastic thyroid lesion in a male patient carrying an MCT8 mutation and papillary hyperplasia in Mct8 knockout mice [48] suggest that MCT8 suppresses thyrocyte cell turnover *in vivo*. Furthermore, we have previously reported that MCT8 suppresses the proliferation of JEG-3 (a choriocarcinoma cell line that is MCT8-null) and N-Tera-2 (a neuronal precursor cell line) cells [32] in a T3-independent manner, just as MCT8 decreased the viability of non-proliferating primary term cytotrophoblast. Interestingly, the EVT-like cell line, SGHPL-4, was unaffected by changes in MCT8 expression and such a contrast suggests that MCT8 regulation of cell viability is a cell-specific effect. Since primary cytotrophoblast do not proliferate in culture, altered cell viability cannot be accounted for by changes in cell proliferation or by an activation of the caspase cascade. MCT8 may instead influence parallel pathways that are caspase-independent leading to increased cell death. How MCT8 affects cell viability independently of T3 is unknown. Investigations into the ability of MCT8 to transport compounds other than THs, have been negative [12], [49]. Alternatively, MCT8 may affect cell viability independently of its transporter function. Regardless of the mechanism responsible, the adverse effect of MCT8 on the viability of cytotrophoblast cells is consistent with our hypothesis that the increased placental expression of MCT8 in IUGR pregnancies may contribute to the pathophysiology of decreased placental size and, hence, fetal size [6], [23], [24].

In mice, we did not observe any significant changes in the expression of a proliferative marker when we compared whole placental homogenates from wild-type with that from Mct8 knockout fetuses. Counting the number of cells within the Lz also revealed no changes in trophoblast (or endothelial) cell number with Mct8 knockout. Only male conceptuses in mice were studied. It is currently unclear whether the discrepancy in MCT8 effects upon cell viability in human trophoblast *in vitro* and upon trophoblast cell number in mouse placenta *in vivo* is due to compensatory mechanisms that may occur *in vivo* or due to species differences. Investigation of placentae from human pregnancies where the fetus carries a MCT8 mutation would be critical in answering this question.

Against a uniform maternal genotype (heterozygous female carriers Mct8^+/−^) in mice and amongst male conceptuses Mct8 knockout resulted in a 25% reduction in fetal to placental weight ratio compared to wild-type, which is indicative of reduced placental efficiency [50]. Placental efficiency is influenced by environmental conditions during pregnancy, as well as by the expression of different genes [51], [52]. For instance, *Phlda2,* a maternally-expressed imprinted gene, is one of the best studied genes which has been strongly associated with birthweight in humans. In mice, *Phlda2* knockout results in increased placental weight with no change or a slight decrease in fetal weight and a 23% reduction in the fetal to placental weight ratio in the mutants compared to wild-type littermates [51], which is comparable to the magnitude of effect on fetal to placental weight ratio observed in our Mct8 model. A reduction in fetal to placental weight ratio may reflect a compensatory mechanism whereby a malfunctioning placenta enlarges to preserve adequate transplacental transport and normalize fetal growth. However, stereohistological analysis of the placenta revealed that the volume fraction of the Lz, which is responsible for transplacental transport in the mouse, was in fact, lower with Mct8 knockout. Therefore, it is unlikely that the difference in the fetal to placental weight ratio reflects an adaptation to optimize maternal-fetal exchange. Instead, there was a general trend of increased volumes of every placental region with Mct8 knockout suggestive of a generalized increase in placental cell growth and an opposite trend of reduced fetal growth at E18.5, which only became significantly different when expressed as a ratio. This suggests that Mct8 knockout has subtle and different effects upon placental and fetal growth, and that differences in fetal to placental weight ratios cannot be explained by isolated effects on trophoblast cell growth.

Our data demonstrate that MCT8 makes a significant contribution to TH transport by human placental cells. Furthermore, changes in the expression of MCT8 affect the two major human trophoblast cell types differently and via both T3-dependent (invasion) and T3-independent (cell viability) mechanisms. However, findings in Mct8 knockout mice show no significant differences in placental cell growth although a reduced fetal to placental weight ratio does suggest subtle effects on overall fetoplacental growth. It is possible that compensatory mechanisms exist *in vivo* that attenuate the effects of Mct8 knockout. However, we cannot exclude the possibility that Mct8 may have different effects on human and mouse placental cells.

## Supporting Information

Figure S1 **Efficiency of MCT8 silencing and over-expression in the EVT-like cell line, SGHPL-4.** **A**: Knock-down of endogenous MCT8 mRNA expression in SGHPL-4 cells was assessed by quantitative RT-PCR. Bars represent average of six experiments +SEM. The mean expression in cells treated with control siRNA was given the arbitrary value of one. Statistically significant differences are indicated by ***P<0.001. **B**: Changes in overall MCT8 protein expression following MCT8 silencing were assessed by western blotting. Whole cell protein lysates (70 µg) of SGHPL-4 cells were probed with rabbit anti-MCT8 antibody [43] followed by secondary anti-rabbit antibody conjugated with HRP. The expression of β-actin was assessed to control for sample loading. **C**: Representative experiment showing the percentage of SGHPL-4 cells that were successfully transfected with HA-tagged MCT8 as assessed by flow cytometry. The cells were probed with a mouse anti-HA antibody (1∶50; Cell Signalling) followed by a secondary antibody labelled with green-fluorescent Alexa Fluor 488 dye (1∶1,000; Invitrogen). Cells transfected with vector only (VO) were used as negative control. **D**: Changes in overall MCT8 protein expression following MCT8 over-expression assessed by western blotting.
(TIFF)

Figure S2 **Efficiency of MCT8 silencing and over-expression in primary term cytotrophoblast cells.** **A**: Knock-down of endogenous MCT8 mRNA expression in cytotrophoblast cells was assessed by quantitative RT-PCR. Bars represent average of three experiments +SEM. The mean expression in cells transfected with control siRNA was given the arbitrary value of one. Statistically significant differences are indicated by ***P<0.001. **B**: Changes in overall MCT8 protein expression following MCT8 silencing were assessed by western blotting. Whole cell protein lysates (30 µg) were probed with rabbit anti-MCT8 antibody [6] followed by secondary anti-rabbit antibody conjugated with HRP. The expression of β-actin was assessed to control for sample loading. **C**: A representative experiment showing the percentage of cytotrophoblast cells that were successfully transfected with HA-tagged MCT8 as assessed by flow cytometry. The cells were probed with anti-HA antibody followed by secondary antibody labelled with green-fluorescent Alexa Fluor 488 dye. Cells transfected with vector only (VO) were used as negative control. **D**: Changes in overall MCT8 protein expression following MCT8 over-expression assessed by western blotting.
(TIFF)
