# Supplementary figures and images for: Monocarboxylate Transporter 8 Modulates the Viability and Invasive Capacity of Human Placental Cells and Fetoplacental Growth in Mice
Source: PLoS One. 2013 Jun 12;8(6):e65402. doi: 10.1371/journal.pone.0065402 (PMC3680392; doi:10.1371/journal.pone.0065402)

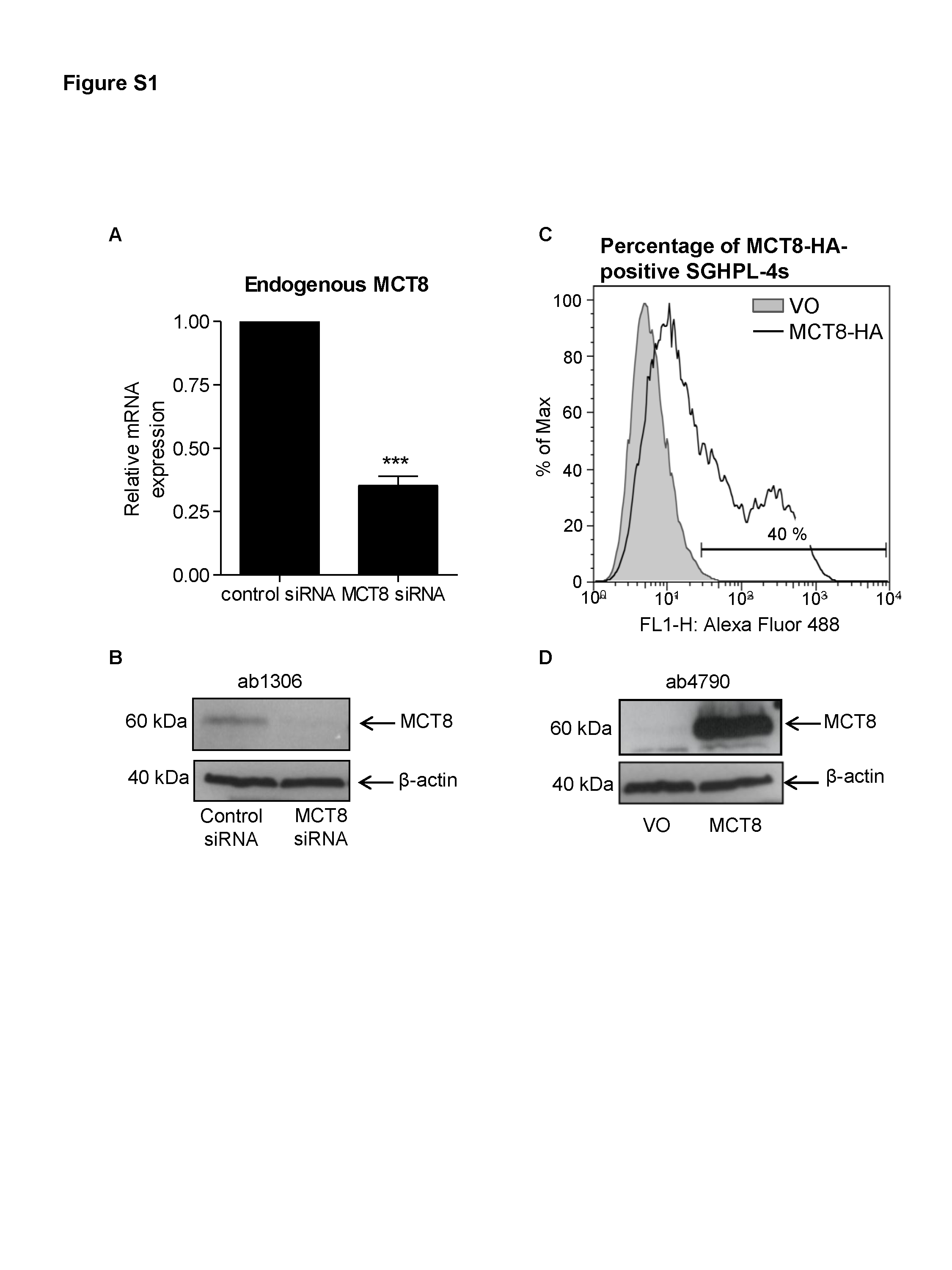

Supplement: Figure S1 — Efficiency of MCT8 silencing and over-expression in the EVT-like cell line, SGHPL-4. A: Knock-down of endogenous MCT8 mRNA expression in SGHPL-4 cells was assessed by quantitative RT-PCR. Bars represent average of six experiments +SEM. The mean expression in cells treated with control siRNA was given the arbitrary value of one. Statistically significant differences are indicated by ***P<0.001. B: Changes in overall MCT8 protein expression following MCT8 silencing were assessed by western blotting. Whole cell protein lysates (70 µg) of SGHPL-4 cells were probed with rabbit anti-MCT8 antibody [43] followed by secondary anti-rabbit antibody conjugated with HRP. The expression of β-actin was assessed to control for sample loading. C: Representative experiment showing the percentage of SGHPL-4 cells that were successfully transfected with HA-tagged MCT8 as assessed by flow cytometry. The cells were probed with a mouse anti-HA antibody (1∶50; Cell Signalling) followed by a secondary antibody labelled with green-fluorescent Alexa Fluor 488 dye (1∶1,000; Invitrogen). Cells transfected with vector only (VO) were used as negative control. D: Changes in overall MCT8 protein expression following MCT8 over-expression assessed by western blotting. (TIFF) [file pone.0065402.s001.tiff]

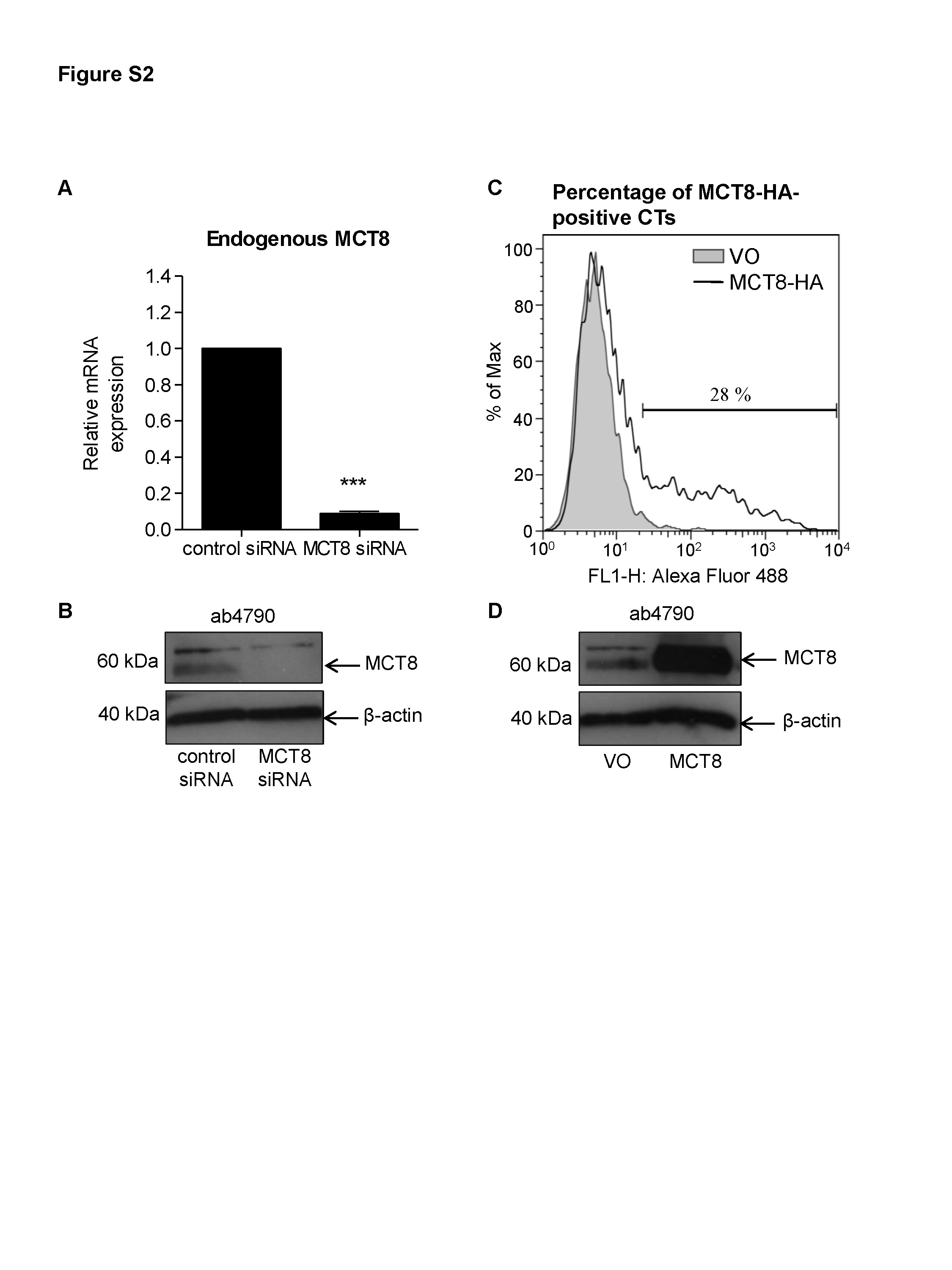

Supplement: Figure S2 — Efficiency of MCT8 silencing and over-expression in primary term cytotrophoblast cells. A: Knock-down of endogenous MCT8 mRNA expression in cytotrophoblast cells was assessed by quantitative RT-PCR. Bars represent average of three experiments +SEM. The mean expression in cells transfected with control siRNA was given the arbitrary value of one. Statistically significant differences are indicated by ***P<0.001. B: Changes in overall MCT8 protein expression following MCT8 silencing were assessed by western blotting. Whole cell protein lysates (30 µg) were probed with rabbit anti-MCT8 antibody [6] followed by secondary anti-rabbit antibody conjugated with HRP. The expression of β-actin was assessed to control for sample loading. C: A representative experiment showing the percentage of cytotrophoblast cells that were successfully transfected with HA-tagged MCT8 as assessed by flow cytometry. The cells were probed with anti-HA antibody followed by secondary antibody labelled with green-fluorescent Alexa Fluor 488 dye. Cells transfected with vector only (VO) were used as negative control. D: Changes in overall MCT8 protein expression following MCT8 over-expression assessed by western blotting. (TIFF) [file pone.0065402.s002.tiff]
